# The association between serum chloride levels and chronic kidney disease progression: a cohort study

**DOI:** 10.1186/s12882-020-01828-3

**Published:** 2020-05-06

**Authors:** Minesh Khatri, Joshua Zitovsky, Dale Lee, Kamal Nayyar, Melissa Fazzari, Candace Grant

**Affiliations:** 1 Division of Nephrology, Department of Medicine, NYU Winthrop Hospital, 200 Old Country Rd, Ste 135, Mineola, NY 11501 USA; 2 Department of Biostatistics, University of North Carolina, Chapel Hill, NC USA; 3 Department of Medicine, NYU Winthrop Hospital, Mineola, NY USA; 4 Department of Epidemiology and Population Health, Albert Einstein College of Medicine, Bronx, NY USA

**Keywords:** Chronic kidney disease, Chloride, Epidemiology

## Abstract

**Background:**

Limited data suggest serum chloride levels associate with mortality in heart failure, chronic kidney disease (CKD), and pulmonary arterial hypertension. Randomized trials have also shown that administration of crystalloid intravenous fluids with lower chloride concentration may have better renal outcomes. However, chloride has not been studied longitudinally for CKD progression.

**Methods:**

We used a prospective cohort of subjects with stage 3 and 4 CKD recruited from a nephrology clinic at a single medical center. Linear regression, linear regression with generalized estimating equations, and Cox proportional hazards models were created for outcomes of overall change in estimated glomerular filtration rate (eGFR), longitudinal changes in eGFR, and time to > 30% decline in eGFR, respectively. Baseline chloride was modeled continuously and categorically, and models were adjusted for potential confounders.

**Results:**

Median follow-up was 1.7 years. Baseline median age was 72 years and median eGFR was 35.7 mL/min/1.73m^**2**^. In multivariable analysis, higher serum chloride associated with worsened eGFR decline. Every 1 mEq/L increase in chloride associated with an overall eGFR decline of 0.32 mL/min/1.73m^**2**^ (***p*** = 0.003), while the difference in eGFR decline in the highest quartile of chloride was 3.4 mL/min/1.73m^**2**^ compared to the lowest quartile (***p*** = 0.004). No association between serum chloride and time to 30% decline in eGFR was observed in multivariable analysis (hazard ratio 1.05 per 1 mEq/L increase in serum chloride, ***p*** = 0.103).

**Conclusions:**

In CKD patients, higher serum chloride associated with a modestly steeper rate of eGFR decline, and may be a useful biomarker to predict CKD progression. Further studies are needed to determine causality.

## Background

Chronic kidney disease (CKD) is highly prevalent globally [1] and associated with significant morbidity and mortality [2]. Despite advances in diagnosis and management, it remains an often-progressive disease with complex and uncertain pathophysiology [3]. Moreover, predicting CKD progression is inexact, and there remains an unmet need for identifying novel biomarkers and mechanistic pathways.

Serum chloride is a frequently neglected laboratory value typically only considered during states of metabolic acidosis. However, it has become increasingly clear that chloride is an important driver of numerous homeostatic mechanisms including regulation of renin secretion, tubuloglomerular feedback, blood pressure response, and renal sodium handling [4–7].

Clinically, serum chloride has been gaining traction as a predictor of adverse outcomes in various clinical settings. In two observational studies of patients with acute decompensated heart failure, low serum chloride associated with diuretic resistance and increased mortality [8, 9]. In a study of patients with pulmonary arterial hypertension, serum chloride < 100 mmol/L 6 months after diagnosis associated with increased mortality, independent of serum sodium levels [10]. Hypochloremia also associated with increased risk of in-hospital mortality after ischemic stroke, as well as all-cause and cardiovascular mortality in a broad cohort of hypertensive patients [11, 12]. As for kidney-specific outcomes, after observational data demonstrated an association between hyperchloremia and acute kidney injury (AKI) [13, 14], two recent randomized control trials demonstrated that both critically and non-critically ill patients who received intravenous fluids with higher chloride concentrations had worsened renal outcomes [15, 16]. Finally, in the only longitudinal study in subjects with stable CKD, hypochloremia associated with both mortality and cardiovascular events [17]. However, to our knowledge no study has examined the relationship of serum chloride levels on long-term CKD progression.

In this study, we used a prospective cohort of mostly CKD stage 3 and 4 patients to determine if there was an association between serum chloride levels and CKD progression. Given recent data that higher concentration of chloride in intravenous fluids were detrimental to short term kidney outcomes, we hypothesized that higher serum chloride levels at baseline would associate with increased risk of long-term CKD progression.

## Methods

### Study participants

**Fig. 1:**
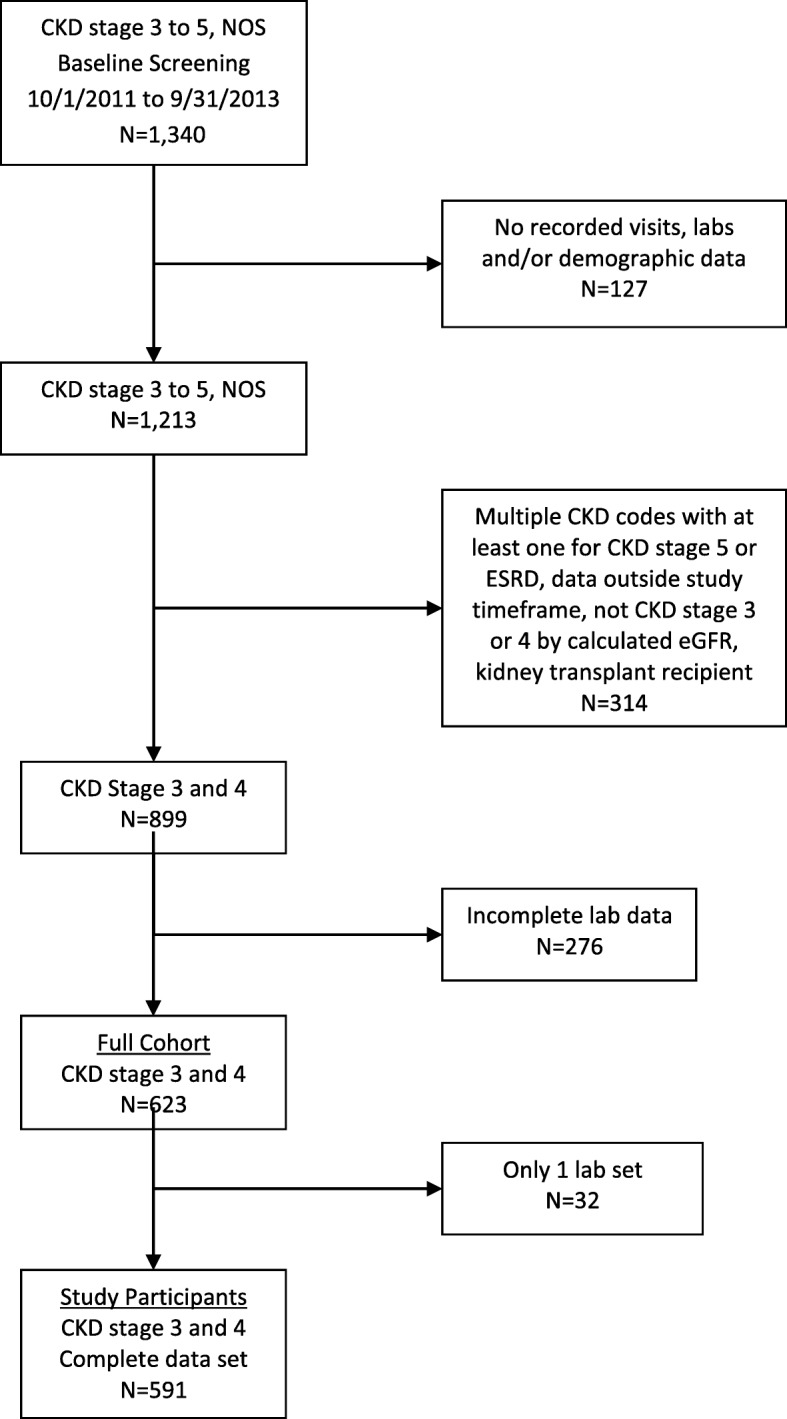
Flowchart of Patients Enrolled in the Study

Participants were enrolled into this observational, prospective, clinic-based cohort study using the electronic health records (EHR) of the patients registered for a visit in the outpatient general nephrology clinic of New York University Winthrop Hospital within the two-year period of October 1, 2011 to September 30, 2013 (Fig. 1, see attached file). The inclusion criteria were subjects who were 18 years or older, were evaluated at least once by a nephrologist in our practice, and had stage 3 or 4 CKD at baseline based on the modification of diet in renal disease (MDRD) formula for eGFR [18]. We created this cohort by initially screening the EHR using the International Classification of Diseases Ninth Revision (ICD-9) codes for CKD stage 3–5 and CKD stage unspecified to identify potential participants (*N* = 1340). Of these, 717 patients were excluded either because 1) there was no actual in-person visit, 2) requisite laboratory data were not available, incomplete, or were outside the study enrollment window, 3) there were multiple ICD-9 codes present with at least one of them being for stage 5 CKD or end stage renal disease, 4) the MDRD eGFR did not place them into CKD stage 3 or 4, 5) they were kidney transplant recipients, or 6) they had incomplete data sets. There were 623 participants ultimately enrolled in the cohort. For this study, participants that only had one visit were eliminated (*N* = 32), thus the sample size was 591. The institutional review board of New York University Winthrop Hospital approved this study.

### Measurement of covariates

Covariates were chosen based on well-established risk factors for CKD and CKD progression, or if there was a significant association with both baseline chloride and change in kidney function in this particular cohort. Demographics (age, gender, and self-identified race and ethnicity), medical comorbidities (hypertension, diabetes, congestive heart failure), and smoking history (never smoked or current/former smoker) were collected at baseline through medical chart review. Insurance status was classified as Medicare, private insurance, or Medicaid/self-insured. Smoking status was classified as current, former, or never smoker based on self-report. Medications including angiotensin converting enzyme inhibitors (ACEIs), angiotensin receptor blockers (ARBs), and diuretics (either loop or thiazide) were recorded at baseline. Laboratory data including serum creatinine, chloride, bicarbonate, phosphate, albumin, calcium, and urine albumin-to-creatinine ratio (ACR) were measured at baseline, with additional data obtained at multiple follow-up visits at the discretion of each patient’s treating physicians. Laboratory tests were run at either NYU Winthrop or an accredited commercial laboratory. The regression equation developed by *Stoycheff,* et al. was used to impute ACR from the protein-to-creatinine ratio when direct measurement of ACR was not available [19]. Enrollment kidney function was calculated using the 4-variable MDRD eGFR equation [18], but was later determined by the CKD-EPI formula for eGFR due to its improved accuracy [20, 21], which are the values used in this analysis. Baseline serum chloride as the primary exposure of interest was modeled both continuously as well as by quartiles within the cohort. Laboratory data were transferred into a research database both manually by co-authors as well as through an automated process when a software interface was available.

### Outcomes

There were three primary outcomes: 1) time to > 30% decline in eGFR; 2) change in eGFR from beginning to the end of the study; 3) change in eGFR longitudinally. Participants were censored for death, dialysis initiation, or kidney transplantation.

### Statistical analyses

The distribution of baseline characteristics was summarized within each quartile of baseline serum chloride using medians with interquartile ranges (IQRs) for continuous variables and frequencies for levels of categorical variables. Spearman’s rank correlation and Cochran-Armitage trend tests were used to test for increasing and decreasing trends across quartiles for values of continuous variables and frequencies of categorical variables, respectively. Kaplan-Meier (KM) survival curves for the time to eGFR decline of at least 30% from baseline were estimated for each quartile of baseline chloride to visualize differences in survival distributions. We presented KM estimates for survival times where at least 10 subjects remained in all four quartile groups. The survival distributions between quartiles were also compared with a logrank test.

We examined the association between serum chloride and CKD progression in three ways: 1) Cox proportional hazards models for the time to first decline of 30% or greater in eGFR from baseline; 2) linear regression models for overall change in eGFR from the baseline visit to the last follow-up visit; and 3) generalized estimated equations (GEE) for eGFR change between visits. For Cox and linear regression models, baseline chloride was modeled both continuously and categorically as quartiles. We also examined the effect of chloride in unadjusted, demographic-adjusted (including age, sex, race and insurance), and fully-adjusted (including laboratory values, medications, and comorbidities) models, where covariates were all measured at baseline. Linear models additionally controlled for study length for all models. Finally, we tested whether there were linear trends between chloride quartile groups and the responses of interest by modelling quartile group as an ordinal variable.

We examined the effect of chloride on eGFR changes between visits in unadjusted, demographic-adjusted and fully-adjusted models as well, but used chloride as a continuous variable only. For each visit, the covariates were those belonging to that visit, and the outcome was eGFR at the next visit. For all models, including unadjusted models, we additionally controlled for the time difference between visits, as well as eGFR of the previous visit. Thus, our models measured the effect of chloride on the rate of eGFR change by the next visit. Covariates that were not laboratory variables, including demographics, medications and comorbidities, were only measured at baseline. For the purposes of this analysis, we assumed that these variables remained the same as baseline for each visit. We used an independent working correlation structure with sandwich estimates for the standard errors. Exchangeable and autoregressive correlation structures did not lead to an improvement in model fit as measured by the quasi-Akaike Information Criterion (QIC), and covariance estimates for visits within the same subject were very close to zero and statistically insignificant. For all GEE, linear and Cox models, we tested for interactions between chloride and bicarbonate as well as between chloride and diuretic usage.

Two-sided *p*-values less than 0.05 were considered statistically significant. All analyses except for drawing the survival curves and at-risk table, including generating the KM estimates and the number of persons at risk at each time point, were done in SAS version 9.4 (SAS Institute Inc., Cary, NC). KM curves and the at-risk table under them were displayed using R version 3.5.1 (R Core Team, Vienna, Austria) with the help of the following packages: ggplot2 v3.1.1 [22], gridExtra v2.3 [23] and cowplot 0.9.4 [24].

## Results

### Baseline characteristics

The final sample size included 591 participants with a median follow-up of 1.7 years (IQR 1–2.4 years) and mean follow-up of 1.7 years (SD 1.0 years). The median number of data points per participant during the entire study was 4 (IQR 3–5), and the median frequency of data points was 2.6 per year. At baseline, median age was 72 years, 62% were male, 53% were diabetic, 92% were hypertensive, and median eGFR was 35.7 mL/min/1.73m^**2**^. The median change in eGFR from baseline to the last follow-up visit was − 2.5 mL/min/1.73m^**2**^ (IQR -7.1 – 2.4 mL/min/1.73m^**2**^), with a median of 4 data points (IQR 3–5) for each patient. Baseline characteristics stratified by quartiles of chloride are shown in Table 1 (see end of document). With higher quartiles of serum chloride, there was a significant decreasing trend for diuretic use, eGFR, serum bicarbonate, serum albumin, serum calcium, and prevalence of congestive heart failure. With higher quartiles there was also a significant increasing trend for increased albuminuria and prevalence of Hispanic race.

**Table 1:** Baseline Characteristics

Variable	Quartile 1 ***N*** = 115	Quartile 2 ***N*** = 167	Quartile 3 ***N*** = 135	Quartile 4 ***N*** = 174	***P***-value
**Chloride range (mEq/L)**	[89–102)	[102–105)	[105–107)	[107–114]	
**Age (years)**	72.0 (17.29)	75.2 (21.99)	73.4 (18.93)	73.2 (16.96)	0.75
**Race**					
White	93 (81)	129 (77)	104 (77)	130 (75)	0.25
Black	13 (12)	26 (16)	19 (14)	26 (15)	0.66
Hispanic	1 (1)	2 (1)	1 (1)	10 (6)	0.006
Other	1 (1)	5 (3)	3 (2)	3 (2)	0.89
Unknown	6 (5)	5 (3)	8 (6)	5 (3)	0.58
**Smoking**					
Current smoker	6 (5)	9 (5)	6 (4)	13 (7)	0.44
Former smoker	48 (42)	77 (46)	69 (51)	74 (43)	0.87
Non-smoker	52 (45)	76 (46)	55 (41)	74 (43)	0.50
Unknown	9 (8)	5 (3)	5 (4)	13 (7)	0.73
**Insurance**					
Medicare	87 (76)	121 (72)	95 (70)	121 (70)	0.25
Private	25 (22)	38 (23)	35 (26)	41 (24)	0.63
Medicaid/Self	3 (3)	8 (5)	5 (4)	12 (7)	0.13
**Female**	46 (40)	63 (38)	44 (33)	70 (40)	0.95
**Uses ACEi/ARB**	74 (64)	98 (59)	87 (64)	125 (72)	0.06
**Uses diuretics**	83 (72)	103 (62)	68 (50)	91 (52)	< 0.001
**Diabetes**	68 (59)	86 (52)	76 (56)	85 (49)	0.17
**Hypertension**	104 (90)	149 (89)	125 (93)	164 (94)	0.12
**CHF**	24 (21)	50 (30)	30 (22)	26 (15)	0.039
**eGFR,** **ml/min per 1.73 m**^**2**^	38.87 (18.4)	34.86 (16.81)	35.91 (19.17)	31.78 (20.22)	< 0.001
**ACR, mg/g**	123 (412.61)	137.02 (454.04)	134.53 (492)	232.2 (691.51)	0.009
**Bicarbonate, mEq/L**	26 (5.5)	26 (5)	24 (4)	23 (6)	< 0.001
**Phosphate, mg/dL**	3.6 (0.8)	3.6 (0.8)	3.6 (0.7)	3.7 (0.7)	0.52
**Albumin, g/dL**	4.2 (0.5)	4.1 (0.4)	4.1 (0.4)	4.0 (0.4)	< 0.001
**Calcium, mg/dL**	9.5 (0.7)	9.3 (0.6)	9.3 (0.7)	9.2 (0.6)	< 0.001

Results are presented as n (%) or median (IQR). *ACEi* Angiotensin-converting enzyme inhibitor, *ARB* Angiotensin receptor blocker, *ACR* Urine albumin-to-creatinine ratio, *CHF* Congestive heart failure

### Analyses

**Fig. 2:**
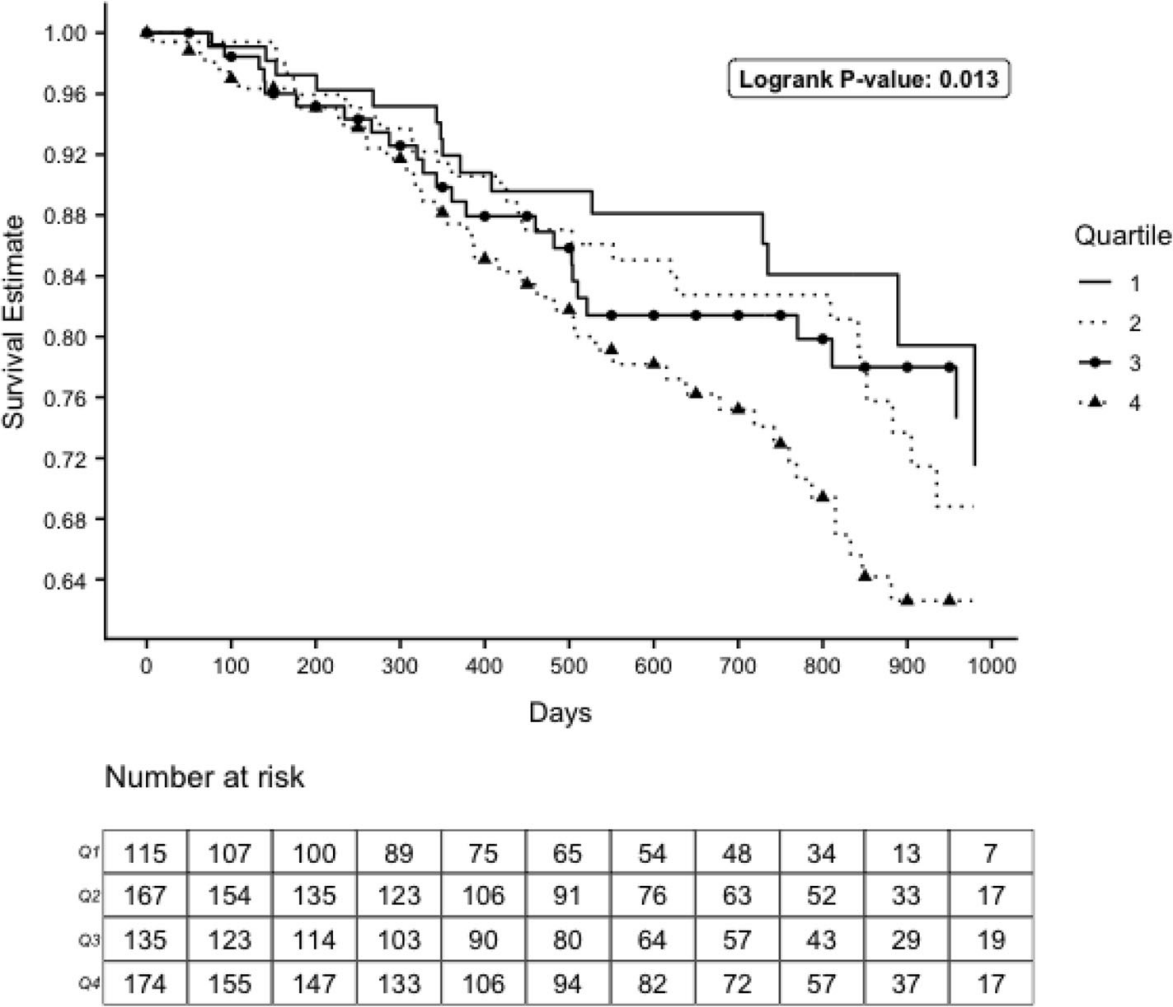
Survival Curve for eGFR Decline > 30%

The Kaplan-Meier survival curve for > 30% eGFR decline is illustrated in Fig. 2 (see attached file). We can see that higher quartiles of chloride have lower estimated survival rates on average, and differences in the survival distributions between the quartiles are statistically significant (logrank *p* = 0.013). A total of 121 participants (20%) reached this threshold of decline by the end of follow-up. While there was a significant association between hazard of > 30% eGFR decline and continuous serum chloride in unadjusted (HR = 1.09; *p*-value < 0.001) and demographic-adjusted models (HR = 1.08; p-value = 0.002), this result became insignificant in fully-adjusted models (HR = 1.05; p-value = 0.10). Similarly, there was no significant association between quartiles of serum chloride at baseline and > 30% decline in eGFR in fully-adjusted models (Table 2).

**Table 2:** Association Between Baseline Serum Chloride and > 30% Decline in eGFR

Cox Model	Hazard-Ratio Estimate	95% C.I.	***P***-value
**Continuous serum chloride (per 1 mEq/L increase)**			
Unadjusted	1.097	(1.045, 1.152)	< 0.001
Demographic adjusted	1.082	(1.029, 1.138)	0.002
Fully adjusted	1.051	(0.99, 1.116)	0.10
**Chloride quartiles (unadjusted)**			
1	Ref	Ref	
2	1.205	(0.662, 2.195)	0.54
3	1.105	(0.596, 2.052)	0.75
4	1.97	(1.135, 3.417)	0.02
Measured ordinally	1.259	(1.053, 1.504)	0.01
**Chloride quartiles (demographic adjusted)**			
1	Ref	Ref	
2	1.188	(0.645, 2.188)	0.58
3	1.129	(0.605, 2.106)	0.70
4	1.683	(0.957, 2.961)	0.007
Measured ordinally	1.182	(0.976, 1.011)	0.07
**Chloride quartiles (fully adjusted)**			
1	Ref	Ref	
2	1.104	(0.588, 2.07)	0.76
3	1.11	(0.581, 2.124)	0.75
4	1.271	(0.676, 2.393)	0.46
Measured ordinally	1.080	(0.877, 1.330)	0.47

In fully adjusted models for overall eGFR change, every 1 mEq/L increase in serum chloride was associated with a 0.323 mL/min/1.73m^2^ (95% confidence interval (CI) -0.538 to − 0.109 mL/min/1.73m^2^, *p*-value = 0.003) overall reduction in eGFR (Table 3).

**Table 3:** Association Between Baseline Serum Chloride and Change in eGFR

Linear Regression Model	Parameter Estimate (in mL/min/1.73 m2)	95% C.I.	***P***-value
**Continuous serum chloride (per 1 mEq/L increase)**			
Unadjusted	−0.315	(− 0.507, − 0.124)	< 0.001
Demographic adjusted	− 0.286	(− 0.479, − 0.093)	0.002
Fully adjusted	− 0.323	(− 0.538, − 0.109)	0.003
**Chloride quartiles (unadjusted)**			
1	Ref	Ref	
2	−0.401	(−2.57, 1.767)	0.72
3	−1.384	(−3.657, 0.889)	0.23
4	−3.215	(−5.366, −1.064)	0.004
Measured ordinally	−1.115	(− 1.783, −0.447)	0.001
**Chloride quartiles (demographic adjusted)**			
1	Ref	Ref	
2	−0.118	(−2.288, 2.051)	0.91
3	−1.101	(−3.371, 1.169)	0.34
4	−2.842	(−5.003, −0.68)	0.01
Measured ordinally	−1.009	(−1.68, −0.339)	0.003
**Chloride quartiles (fully adjusted)**			
1	Ref	Ref	
2	−0.626	(−2.735, 1.484)	0.56
3	−1.696	(−3.927, 0.536)	0.14
4	−3.378	(−5.638, −1.118)	0.004
Measured ordinally	−1.149	(−1.858, −0.44)	0.002

When tested categorically, the association with eGFR decline was stronger with higher quartiles of chloride, with the fourth quartile of chloride associated with an overall eGFR decline of 3.378 mL/min/1.73m^2^ (95% CI − 5.366 to − 1.064 mL/min/1.73m^2^, *p* = 0.004) compared to the first (lowest) quartile. In fully adjusted GEE models based on repeated eGFR measurements, we found that a 1 mEq/L increase in serum chloride was significantly associated with a 0.208 mL/min/1.73m^2^ reduction (95% CI − 0.302 to − 0.114 mL/min/1.73m^2^, *p*-value <  0.001) in eGFR at the next visit (Table 4). While we adjusted for study length in all linear models, we also tested for correlation between study length and rate of eGFR change, which was insignificant (*p* = 0.054, correlation = 0.08). Finally, interactions between serum chloride and diuretic use and serum bicarbonate level were insignificant for all models.

**Table 4:** Association Between Serum Chloride and Change in eGFR at Next Visit

GEE Model	Parameter Estimate (in mL/min/1.73 m2) per 1 mEq/L increase in serum chloride	95% C.I.	***P***-value
Unadjusted	−0.147	(−0.261, − 0.032)	0.01
Demographic	−0.138	(− 0.250, − 0.023)	0.02
Full	− 0.208	(− 0.302, − 0.114)	< 0.001

## Discussion

In this observational, prospective cohort we found that higher serum chloride associated with modestly worsened eGFR decline during the course of 1.7 years of median follow-up, independent of other well-established risk factors for CKD progression. Serum chloride was associated with > 30% decline in eGFR in demographic adjusted models, but the results were attenuated and insignificant in fully adjusted models. There was no clear threshold effect, with continuous models of serum chloride demonstrating an association with eGFR decline for every 1 mEq/L increase.

Our study adds to the growing body of evidence implicating a role for chloride in human disease. Several recent observational studies have focused on heart failure. In one analysis of patients admitted for acute decompensated heart failure, lower levels of serum chloride were independently associated with long-term mortality [8]. In another acute heart failure cohort, baseline hypochloremia associated with impaired diuresis, and persistent hypochloremia with increased mortality [9]. Low serum chloride levels also associated with higher mortality in chronic heart failure [22, 23]. Two additional observational studies found independent relationships between baseline hypochloremia and increased in-hospital mortality in patients with acute stroke, as well as all-cause mortality in hypertensive patients followed longitudinally [11, 12].

While these observational studies yielded adverse outcomes associated with hypochloremia, ours showed possible worsened outcomes with higher serum chloride levels. This discordance may be due to differences in study design and outcomes, as prior studies were conducted in different populations where there may be more hemodynamic and volume issues, and a stronger role for diuretics which may have impacted chloride levels and outcomes. These studies also did not specifically focus on kidney function, where chloride has important physiologic roles. For instance, previous observational studies found associations between hyperchloremia, chloride-liberal intravenous fluids, and acute kidney injury (AKI) [13, 24]. These relationships were later confirmed in two large randomized control trials that showed superior acute renal outcomes with balanced crystalloids versus normal saline [15, 16]. In the one observational study with serum chloride that focused on a cohort of CKD participants, hyperchloremia associated with worsened eGFR at baseline (22.6 mL/min/1.73m^2^ vs 36.3 mL/min/1.73m^2^, in the highest versus the lower quartile of serum chloride, respectively), which correlates with our findings, although this study did not examined changes in eGFR longitudinally [17].

Whether or not a possible association seen in this study between chloride and long-term change in eGFR is causal or translates into meaningful clinical outcomes requires further investigation and replication, however there are possible physiologic mechanisms that may link these findings. For instance, plasma chloride, not sodium, experimentally reduced renal blood flow in denervated canine kidneys [25]. In the same study, the importance of the anion accompanying sodium was highlighted whereby both ammonium chloride and sodium chloride caused reductions in renal blood flow and glomerular filtration rate, while infusion of sodium bicarbonate caused renal vasodilation [25]. Hyperchloremia may also lead to increased interstitial chloride concentration with a possible direct effect on afferent arteriolar vasoconstriction that may be independent of its effects on tubuloglomerular feedback [26]. Data in human and animal models suggest chloride is an important determinant of sodium sensitive hypertension [5], and that chloride (but not sodium or water) accumulation in skin is associated with salt sensitive hypertension in experimental mouse models [27]. Dietary sodium intake accompanied by non-chloride anions (such as phosphate) have been demonstrated to be less likely to raise blood pressure compared to dietary sodium intake accompanied by chloride [28]. Our analysis also showed that hyperchloremia associated with reduced serum albumin. Although we adjusted for serum albumin in multivariate models, it is possible that lower serum albumin may reflect other unmeasured confounders, including inflammation, that may impact kidney function. Some studies have suggested an increase in inflammation associated with hyperchloremic acidosis and saline-rich intravenous fluids in experimental sepsis models [29]. It is unclear whether some of these pathways, which may play a role in AKI, have any role in CKD.

Another possible explanation for the observed association involves metabolic acidosis, since higher serum chloride levels are often seen concurrently with metabolic acidosis, which is associated with increased risk of CKD progression [30, 31]. Metabolic acidosis in CKD usually becomes more common once GFR falls below 40 mL/min, when total ammonium excretion starts to decrease despite the increase in per-nephron ammonium excretion. The increased ammonium excretion may eventually become maladaptive, leading to complement activation promotion of interstitial inflammation [32]. While there was a correlation in our study between higher serum chloride and lower serum bicarbonate, we adjusted for serum bicarbonate in multivariable analyses where change in eGFR was the outcome, and chloride was still a significant independent predictor. Moreover, we also tested for interactions between serum chloride and bicarbonate, which were insignificant, suggesting that serum bicarbonate does not modify the association between serum chloride and eGFR change seen in our study. Nevertheless, it is still possible that metabolic acidosis may be playing a role, as we could not account for factors such as dietary acid load which have been associated with both incident and progressive CKD [33, 34].

There are several strengths to this study including rigorous collection of data, a cohort comprised of a real-world population with chronic kidney disease, meticulous follow-up, several statistical models each adjusted for a broad range of well-established CKD risk factors, and several data points on average for each participant to limit chance fluctuations in eGFR. However, our study also has limitations. First, the median follow-up was only 1.7 years with an overall relatively small change in eGFR, which may have reduced the power to detect > 30% decline in eGFR. Power was not calculated a priori to conducting this analysis. Our study was also too short to determine hard outcomes including end stage renal disease. However, two different methods to test the association with continuous eGFR change both yielded similar results, making the findings more robust. Second, participants were all from a nephrology clinic with established CKD, which may limit generalizability to the general population either with or without CKD. The study population also was skewed older and towards whites with Medicare and private insurance. Finally, causality cannot be determined from an observational study and we cannot exclude the possibility that our findings may be related to unmeasured bias and confounding. It is entirely possible that associations between serum chloride and long-term changes in eGFR are hemodynamic, and may not reflect underlying kidney disease progression. However, we adjusted for diuretics, ACE inhibitors/ARBs, and heart disease in our final models, which are most likely to cause hemodynamic changes, and there was still a significant association between serum chloride and change in eGFR.

## Conclusion

In conclusion, in a prospective cohort of men and women with CKD, we demonstrated that higher serum chloride is significantly associated with a modest decrease in eGFR independent of other well-established CKD risk factors. Future studies should examine and confirm this relationship over longer periods of follow-up, and in more diverse populations including those without preexisting CKD. Further research is also needed to determine causality, and possible underlying pathophysiology.

## Supplementary information

**Additional file 1: Table S1.** Baseline Characteristics. Results are presented as mean (standard deviation).

## Data Availability

The datasets used during the current study are available from the corresponding author on reasonable request.

## Abbreviations

CKD: Chronic kidney disease
AKI: Acute kidney injury
eGFR: Estimated glomerular filtration rate
ACE inhibitor: Angiotensin converting enzyme inhibitor
ARB: Angiotensin receptor blocker
CHF: Congestive heart failure
ACR: Urine albumin-to-creatinine ratio
ESRD: End stage renal disease
NOS: Not otherwise specified
