# Supplementary material for: The association between serum chloride levels and chronic kidney disease progression: a cohort study
Source: BMC Nephrol. 2020 May 6;21:165. doi: 10.1186/s12882-020-01828-3 (PMC7203999; doi:10.1186/s12882-020-01828-3)
Supplement: Supplementary file 1 — Additional file 1: Table S1. Baseline Characteristics. Results are presented as mean (standard deviation). [file 12882_2020_1828_MOESM1_ESM.docx]

# Supplemental Table 1: Baseline Characteristics

Results are presented as mean (standard deviation)

| **Variable** | **Quartile 1**  **N=115** | **Quartile 2**  **N=167** | **Quartile 3**  **N=135** | **Quartile 4**  **N=174** | **P-value** |
| --- | --- | --- | --- | --- | --- |
| **Chloride range (mEq/L)** | [89-102) | [102-105) | [105-107) | [107-114] |  |
| **Age (years)** | 70.2 (13.3) | 71.9 (13.9) | 71.2 (13.2) | 70.4 (14.3) | 0.803 |
| **eGFR,**  **ml/min per 1.73 m^2^** | 38.2 (12.2) | 35.2 (12.3) | 35.6 (11.9) | 32.4 (11.8) | < 0.001 |
| **ACR, mg/g** | 572.6 ( 1065.4) | 633.3 (1586.1) | 599.7 (1275.6) | 797.5 (1605.1) | 0.203 |
| **Bicarbonate, mEq/L** | 26 (4) | 26 (4) | 24 (3) | 22 (4) | <0.001 |
| **Phosphate, mg/dL** | 3.7 (0.7) | 3.6 (0.6) | 3.6 (0.6) | 3.7 (0.7) | 0.438 |
| **Albumin, g/dL** | 4.1 (0.4) | 4.1 (0.4) | 4.1 (0.3) | 4.0 (0.4) | <0.001 |
| **Calcium, mg/dL** | 9.5 (0.5) | 9.3 (0.5) | 9.4 (0.5) | 9.2 (0.5) | <0.001 |
